# Characteristics and prognosis of primary treatment-naïve oral cavity squamous cell carcinoma in Norway, a descriptive retrospective study

**DOI:** 10.1371/journal.pone.0227738

**Published:** 2020-01-16

**Authors:** Inger-Heidi Bjerkli, Olav Jetlund, Gunnhild Karevold, Ása Karlsdóttir, Ellen Jaatun, Lars Uhlin-Hansen, Oddveig G. Rikardsen, Elin Hadler-Olsen, Sonja E. Steigen

**Affiliations:** 1 Department of Otorhinolaryngology, University Hospital of North Norway, Tromsø, Norway; 2 Department of Medical Biology, UiT The Arctic University of Norway, Tromsø, Norway; 3 Department of Otorhinolaryngology, Head and Neck Surgery, Division of Head, Neck and Reconstructive Surgery, Oslo University Hospital—Rikshospitalet, Oslo, Norway; 4 Department of Oncology, Haukeland University Hospital, Bergen, Norway; 5 Department of Otorhinolaryngology, St. Olavs University Hospital, Trondheim, Norway; 6 Norwegian University of Science and Technology, Trondheim, Norway; 7 Department of Pathology, University Hospital of North Norway, Tromsø, Norway; 8 Department of Clinical Dentistry, UiT The Arctic University of Norway, Tromsø, Norway; University of Cincinnati College of Medicine, UNITED STATES

## Abstract

**Objectives:**

Incidence of oral cavity squamous cell carcinomas is rising worldwide, and population characterization is important to follow for future trends. The aim of this retrospective study was to present a large cohort of primary oral cavity squamous cell carcinoma from all four health regions of Norway, with descriptive clinicopathological characteristics and five-year survival outcomes.

**Materials and methods:**

Patients diagnosed with primary treatment-naïve oral cavity squamous cell carcinomas at all four university hospitals in Norway between 2005–2009 were retrospectively included in this study. Clinicopathological data from the electronic health records were compared to survival data.

**Results:**

A total of 535 patients with primary treatment-naïve oral cavity squamous cell carcinomas were identified. The median survival follow-up time was 48 months (range 0–125 months) after treatment. The median five-year overall survival was found to be 47%. Median five-year disease-specific survival was 52%, ranging from 80% for stage I to 33% for stage IV patients. For patients given treatment with curative intent, the overall survival was found to be 56% and disease-specific survival 62%. Median age at diagnosis was 67 years (range 24–101 years), 64 years for men and 72 years for women. The male: female ratio was 1.2. No gender difference was found in neither tumor status (p = 0.180) nor node status (p = 0.266), but both factors influenced significantly on survival (p<0.001 for both).

**Conclusions:**

We present a large cohort of primary treatment-naïve oral cavity squamous cell carcinomas in Norway. Five-year disease-specific survival was 52%, and patients eligible for curative treatment had a five-year disease-specific survival up to 62%.

## Introduction

Oral cavity cancer (OCC) is the most common subtype of head and neck (HN) cancer [1], and includes cancers in the mobile tongue (anterior 2/3 of the tongue), floor of mouth, buccal and labial mucosa, upper and lower gingiva and alveolar mucosa, retromolar trigone, and hard palate [2–4]. The mobile tongue is the most common site for OCC, accounting for up to 50% of the cases [5–7].

In 2012 the global incidence of OCC was estimated to 275 000 [1], and is steadily rising worldwide. According to global cancer statistics in 2018, the estimated incidence of OCC together with lip location was found to be around 355 000 [8]. However, there is geographical variation; as many as 25% of all cancers in high-risk countries in South-East Asia are oral carcinoma [8, 9]. In Europe, the incidence is higher in Southern and Central/Eastern parts compared to Northern and Western parts [9–11]. For cancer of the tongue, there is a trend of increasing incidence in the Nordic countries as well as in the United States [5, 12–14]. The now recognized importance of HPV infection in developing oropharyngeal cancer has stressed the importance of distinguishing OCC from oropharyngeal cancers [1, 15]. HPV has been found to be uncommon in OCC [16, 17]. The incidence of oropharyngeal cancers was estimated globally to be around 93 000 cases in 2018 [8].

Squamous cell carcinoma (SCC) accounts for more than 90% of malignant neoplasms of the oral cavity [11, 15, 18], and is classified by the TNM system according to primary tumor size (T), regional lymph node spread (N), and distant metastasis (M) [3, 4].

Tobacco smoking, betel chewing, and excessive alcohol drinking are major risk factors, though the habit of betel nut chewing is a factor mainly in Asia [9, 18–20]. Poor dental health is also considered to be a risk factor [20–23]. Some patients experience recurrence or risk of second primary tumors [24–27].

Primary surgery is the preferred treatment for oral cavity squamous cell carcinoma (OCSCC) in most institutions when the tumor is regarded resectable, with or without reconstruction and neck dissection. Postoperative adjuvant radiation therapy (RT) is often necessary, whereas chemotherapy is seldom used, except sometimes for advanced stages [28–34]. The treatment should be decided by a multidisciplinary team (MDT) [35]. In lack of a national treatment protocol for HN cancer, management of OCSCC in Norway usually follows the protocol published by the Danish Head and Neck Cancer Group (DAHANCA) [34].

Five-year survival rate for OCSCC is approximately 50% for most countries [9, 11, 36]. Despite earlier detection and more treatment options, survival rate has not improved more than three to five percent over the last decades [11, 15]. The Surveillance, Epidemiology, and End Result program (SEER) database has published five-year relative survival rate of 66% for tongue, and 53% for the floor of mouth in the period 2009–2015 [37].

The epidemiological and survival data for OCC are hampered with uncertainty as many studies report results from small patient cohorts, often selected from a single or a referral hospital, or a small region. Furthermore, some studies include only patients treated with curative intention, or unfortunately merge patients with cancers of various subsites of the HN region [5–7, 12, 16].

The aim of this retrospective study was to present a large cohort of OCSCC, from all four health regions of Norway, with descriptive clinicopathological characteristics and five-year survival outcomes. All patients were diagnosed with primary treatment-naïve OCSCC in the period 2005–2009, and the results were evaluated against comparable cohorts.

## Materials and methods

### Data collection process

The Norwegian Oral Cancer (NOROC) study is a retrospective study that includes patients diagnosed with primary treatment-naïve OCSCC in the four university hospitals in Norway between January 1^st^ 2005 through December 31^st^ 2009. In Norway, management of OCC is centralized to university hospitals of Rikshospitalet (Oslo), Haukeland (Bergen), St. Olavs (Trondheim) and North Norway (Tromsø), where Rikshospitalet in Oslo (The National Hospital) also is regarded as a tertiary referral hospital. Patients were identified by searching for the relevant International Statistical Classification of Diseases and Related Health Problems 10^th^ Revision (ICD-10) codes in the electronic health records (EHR) of these hospitals, as well as by searching the pathology archives for cancers with topographic systematically organized computer-processable collection of medical terms providing codes (SNOMED) coding T51 and T53. The patients diagnosed during this period were classified according to TNM 5^th^ Edition 2005 UICC [3].

We included patients with the relevant ICD-10 classification codes C02-C06 [38], which refer to cancers in the buccal and labial mucosa, upper and lower gingiva and alveolar mucosa, hard palate, mobile tongue, and floor of mouth. We excluded ICD-10 codes C05.1 and C05.2 which are regarded as oropharyngeal sites, and cancer of the external upper or lower lip (vermilion), because these almost exclusively arise in the lower lip and are more likely to act as skin cancer [15]. Tumors with different histopathology than SCC were also excluded, as well as patients with HN second primaries or previous cancer treatment. Approximately 27% of the patients had been incorrectly coded; the majority of these had oropharyngeal cancer, and were excluded.

### Extracting clinical data

Anonymized clinical data were recorded in a web-based Case Report Form (CRF). The last day of follow-up was June 1^st^ 2015 when all patients had been followed throughout a minimum of five years after end of treatment. The patient EHRs were screened from date of diagnosis until date of death, or from date of diagnosis until last day of follow-up. Recording was done by experienced clinicians (IHB, OJ, GK, EJ and ÁK). Relevant patient data, ICD-10 diagnosis, TNM classification, treatment, and follow-up were registered. Since these patients were diagnosed between 2005–2009, the stage classification was done according to AJCC 6^th^ edition 2002 [15].

In the TNM classification system, the term TX, NX, and MX can be used in cases where the primary tumor, regional lymph nodes, or distant metastasis cannot be assessed [3, 4, 15]. For this reason, some tumors lacked T, N, or M status. However, a T4 tumor could be staged without knowledge of the N and M status, since it automatically classifies as a stage IV tumor. This was also true with N status ≥N2, and M1. In this study we pooled stage IVA, IVB, and IVC into Stage IV.

The study was approved by the Institutional Review Board of Northern Norwegian Regional Committee for Medical Research Ethics (REK Nord) giving validated approval for all four hospitals (Protocol number REK Nord; 2013/1786 and 2015/1381). REK Nord waved the need for the patients still alive to have the opportunity to opt-out when they were informed about the project. The information-consent letter was approved by REK Nord before being sent out to the patients still alive. This study was planned retrospectively and approved in 2014, five to ten years after the patients had had their diagnosis and treatment. Patients were informed they could withdraw from the study without concern. The letter was sent to patients when the inclusion stage of research commenced between August 2015 and February 2017. The patient information-consent letter was sent to those still alive giving them the option to opt-out of the study. This was executed by the principal investigator, who received the list over patients still alive from one of the co-investigators who was the only one with access to the patient’s identifying data. The address used was the latest address given in the EHR. No letters were returned from patients or postal services. Three patients contacted the principal investigator to confirm they were agreeing to participate, no one contacted to opt-out. Cause of death was acquired from Norwegian Cause of Death Registry.

### Categorical grouping

Patients were divided into groups based on age at time of diagnosis; 51–60 years, 61–70 years, and 71–80 years. Those younger than 50 and older than 80 were few and thus pooled in a younger (≤50 years) and an older (≥81 years) age group. We also organized the patients according to an indicator called Integrated Risk Factor (IRF) based on extent of tobacco and alcohol consumption as previously described by Rikardsen et al. [16].

Patients with alcohol consumption recorded as seldom in the EHRs were classified as “light drinkers” (≤ 1 times weekly), whereas those with consumption denoted as current, moderate, heavy, or former alcoholic abuse were classified as “drinkers” (> 1 times weekly or daily) [39, 40]. Based on information from the EHR, dental status was categorized as good (no dental treatment needed), need of dental therapy before start of treatment, or edentulous [41]. Cancer treatment described in the EHR was categorized into different groups/combinations of treatment modalities. Palliative treatment and treatment vaguely described, were pooled. Level of education was poorly described in the EHRs and could not be used to describe socioeconomic status.

### Statistical analyses

The correlation between gender and different variables was evaluated using Spearman bivariate correlation (2-tailed) and bootstrapping at 95% confidence interval (CI), as shown in Tables 1–3. For evaluating survival, Cox regression allowed us to report significance, hazard ratio (HR), and 95% CI after bootstrapping as shown in Tables 4 and 5. Results were considered to be significant at p<0.05. For survival the variables significant in univariate analysis were analyzed for multicollinearity (VIF), applying linear regression, testing independent variables against a dependent variable. VIF values <2 were regarded to indicate no multicollinearity. The variables with limited data (few in number), were excluded from calculations because of risk of sparse-data bias [42–44]. Kaplan-Meier (Log Rank) was used to construct survival analyses plot. For survival analysis the definitions used were overall survival (OS) and disease-specific survival (DSS); the latter was equivalent to cause-specific survival [45]. All statistical analyses were performed with IBM Statistical Package for the Social Sciences (SPSS) version 25.

**Table 1:** Clinicopathological characteristics of 535 primary oral cavity squamous cell carcinomas 2005–2009.

	Male n (%)	Female n (%)	(r_s_)	(CI 95%)	p
**Variable**	294 (55)	241 (45)			
Age, median (range)	64 (25–101)	72 (24–96)			
**Age groups**					
≤50	31 (10.5)	19 (7.9)			
51–60	72 (24.5)	36 (14.9)			
61–70	108 (36.7)	55 (22.8)	0.245	(0.162–0.325)	<0.001
71–80	54 (18.4)	66 (27.4)			
>80	29 (9.9)	66 (27.0)			
**Primary site**					
Mobile tongue	142 (48.3)	98 (40.7)			
Gingival/alveolar	46 (15.6)	61 (25.3)			
Floor of mouth	69 (23.5)	33 (13.7)	0.062	(-0.026–0.147)	0.154
Cheek/bucca/retromolar	35 (11.9)	44 (18.3)			
Hard palate	2 (0.7)	5 (2.1)			
**Tumor status**					
T1	65 (22.1)	46 (19.1)			
T2	100 (34.0)	73 (30.3)			
T3	29 (9.9)	29 (12.0)	0.059	(-0.030–0.141)	0.180
T4	92 (31.3)	85 (35.3)			
Unknown*	8 (2.7)	8 (3.3)			
**Lymph node status**					
N0	186 (63.3)	143 (59.3)			
N1	34 (11.6)	23 (9.5)			
N2	53 (18.1)	56 (23.2)	0.050	(-0.039–0.136)	0.266
N3**	4 (1.4)	1 (0.4)			
Unknown*	17 (5.8)	18 (7.5)			
**Stage of disease**					
Stage I	61 (20.7)	40 (16.6)			
Stage II	75 (25.5)	51 (21.2)			
Stage III	35 (11.9)	28 (11.6)	0.089	(-0.004–0.172)	0.043
Stage IV	112 (38.1)	111 (46.1)			
Unknown*	11 (3.7)	11 (4.6)			

* Unknown data were not included in the calculations.

**Not included in calculations because of risk of sparse-data bias.

r_s_ = Spearman rank correlation, rho.

**Table 2:** Risk factors for 535 patients with primary oral cavity squamous cell carcinoma in Norway 2005–2009.

	Male n (%)	Female n (%)	(r_s_)	(CI 95%)	p
**Variable**	294 (55)	241 (45)			
**Smoking**					
Never	41 (13.9)	82 (34.0)			
Current	169 (57.5)	97 (40.2)	-0.230	(-0.317–0.137)	<0.001
Former	70 (23.8)	37 (15.4)			
Unknown*	14 (4.7)	25 (10.4)			
**Alcohol comsumption**					
Never (Non-drinker)	12 (4.1)	36 (14.9)			
≤1 times weekly (Light drinker)	42 (14.3)	55 (22.8)	-0.405	(-0.501–0.305)	<0.001
>1 times weekly or daily (Drinker)	155 (52.7)	49 (20.3)			
Unknown*	85 (28.9)	101 (41.9)			
**Integrated Risk Factor**					
Non-smoker/Non-drinker	12 (4.1)	32 (13.3)			
Non-smoker/Light drinker	39 (13.3)	41 (17.0)			
Smoker/Non-drinker**	1 (0.3)	5 (2.1)	-0.084	(-0.201–0.036)	0.119
Smoker/Light drinker	15 (5.1)	20 (8.3)			
Smoker/Drinker	138 (46.9)	42 (17.4)			
Unknown*	89 (30.3)	101 (41.9)			
**Dental status**					
Good	68 (21.4)	44 (18.3)			
Needs treatment	158 (53.7)	112 (46.5)	0.119	(0.024–0.206)	0.009
Edentulous	45 (15.3)	64 (26.6)			
Unknown*	28 (9.5)	21 (8.7)			

* Unknown data were not included in the calculations.

**Not included in calculations because of risk of sparse-data bias.

r_s_ = Spearman rank correlation, rho.

**Table 3:** Treatment of 535 primary oral cavity squamous cell carcinoma patients 2005–2009.

	All n (%)	Male n (%)	Female n (%)	(r_s_)	(CI 95%)	p
**Patients**	535 (100)	294 (55.0)	241 (45.0)			
**Treatment intention**						
Curative	427 (79.8)	239 (81.3)	188 (78.0)			
Palliative	26 (4.9)	12 (4.1))	14 (5.8)	0.046	(-0.052–0.141)	0.329
Unknown*	82 (15.3)	43 (14.6)	39 (16.2)			
**Given treatment**						
**Surgery**						
Surgery alone	125 (23.4)	58 (19.7)	67 (27.8)			
Surgery + postop RT	235 (43.9)	147 (50.0)	88 (36.5)			
Preop RT + surgery**	4 (0.7)	2 (0.7)	2 (0.8)	-0.134	(-0.240–0.034)	0.009
Surgery + pre- and postop RT**	1 (0.2)	1 (0.3)	0			
Surgery with chemo**	0	0	0			
Surgery with RT and chemo	21 (3.9)	12 (4.1)	9 (3.7)			
**Non-surgery**						
RT alone	63 (11.8)	34 (11.6)	29 (12.0)			
Chemo alone**	0	0	0			
RT + chemo	23 (4.3)	12 (4.1)	11 (4.6)	0.016	(-0.194–0.241)	0.884
No treatment**	2 (0.4)	1 (0.3)	1 (0.4)			
Unknown*	3 (0.6)	2 (0.7)	1 (0.4)			
Palliative	58 (10.8)	25 (8.5)	33 (13.7)			
**Neck surgery**						
No neck surgery	255 (47.7)	133 (45.2)	122 (50.6)			
Elective neck	76 (14.2)	49 (16.7)	27 (11.2)			
Selective neck	52 (9.7)	30 (10.2)	22 (9.1)	-0.079	(-0.166–0.013)	0.094
Modified radical and radical neck	68 (12.7)	41 (13.9)	27 (11.2)			
Unknown neck surgery*	84 (15.7)	41 (14.0)	43 (17.8)			

* Unknown data were not included in the calculations.

**Not included in calculations because of risk of sparse-data bias.

r_s_ = Spearmans rank correlation, rho.

RT = Radiation therapy

Chemo = Chemotherapy

**Table 4:** Clinicopathological characteristics and five-year overall survival and disease-specific survival.

			OS				DSS	
	OS n (%)	HR	(CI 95%)	p	DSS n (%)	HR	(CI 95%)	p
**Patients**	251 (100)				225(100)			
**Gender**								
Male	137 (55)				122 (54)			
Female	114 (45)	1.022	(-0.206–0.248)	0.853	103 (46)	1.009	(-0.249–0.271)	0.929
**Age groups**								
≤50	35 (70.0)				35 (72.9)			
51–60	65 (57.4)				59 (60.2)			
61–70	88 (54.0)	1.435	(1.299–1.586)	<0.001	81 (61.4)	1.580	(0.338–0.594)	0.001
71–80	48 (40.0)				38 (41.3)			
>80	18 (19.1)				12 (18.5)			
**Primary site**								
Mobile tongue	117 (48.8)				106 (54.4)			
Gingiva/alveolar	49 (45.8)				44 (48.9)			
Floor of mouth	50 (40.9)	1.041	(-0.046–0.121)	0.338	43 (54.4)	1.058	(-0.041–0.152)	0.228
Cheek/bucca/retromolar	33 (41.8)				30 (45.5)			
Hard palate	2 (28.6)				2 (40.0)			
**Tumor status**								
T1	70 (63.1)				66 (72.5)			
T2	95 (54.9)				83 (61.9)			
T3	19 (32.8)	1.401	(0.232–0.437)	<0.001	16 (34.0)	1.531	(0.311–0.553)	<0.001
T4	60 (33.9)				53 (34.6)			
Unknown*	7 (38.9)				7 (70.0)			
**Lymph node status**								
N0	193 (58.7)				174 (65.2)			
N1	21 (36.8)				18 (37.5)			
N2	22 (20.2)	1.825	(0.469–0.741)	<0.001	22 (22.9)	1.929	(0.509–0.816)	<0.001
N3**	0 (0)				0 (0)			
Nx/Unknown*	7 (50.0)				3 (51.3)			
**Stage of disease**								
Stage I	69 (68.3)				65 (80.2)			
Stage II	77 (61.1)				67 (67.7)			
Stage III	29 (46.0)	1.435	(0.261–0.481)	0.001	24 (45.3)	1.665	(1.356–1.729)	0.001
Stage IV	68 (30.5)				62 (32.6)			
Unknown*	8 (36.4)				7 (58.3)			

* Unknown data were not included in the calculations.

**Not included in calculations because of risk of sparse-data bias.

**Table 5:** Risk factors and five-year overall survival and disease-specific survival.

			OS				DSS	
	OS n (%)	HR	(CI 95%)	p	DSS n (%)	HR	(CI 95%)	p
**Patients**	251 (100)				225 (100)			
**Gender**								
Male	137 (55)				122 (54)			
Female	114 (45)	1.022	(-0.206–0.248)	0.853	103 (46)	1.009	(-0.249–0.271)	0.929
**Smoking**								
Never	61 (49.6)				56 (52.8)			
Current	125 (47.0)	0.915	(-0.234–0.055)	0.222	111 (52.4)	0.926	(-0.254–0.110)	0.372
Former	51 (47.7)				45 (51.1)			
Unknown*	12 (32.4)				11 (40.7)			
**Alcohol comsumption**								
Never (Non-drinker)	18 (37.5)				14 (38.9)			
≤1 times weekly (Light- drinker)	49 (50.5)	0.929	(-0.203–0.052)	0.246	48 (55.8)	0.916	(-0.239–0.069)	0.242
>1 times weekly/daily (Drinker)	90 (44.1)				80 (48.5)			
Unknown*	94 (50.5)				83 (56.1)			
**Integrated Risk Factor**								
Non-smoker/Non-drinker	17 (38.6)				12 (37.5)			
Non-smoker/Light-drinker	46 (57.5)	0.969	(-0.067–0.006)	0.081	44 (62.0)	0.965	(-0.081–0.006)	0.091
Smoker/Non-drinker**	3 (50.0)				3 (60.0)			
Smoker/Light drinker	18 (51.4)				18 (56.3)			
Smoker/Drinker	70 (38.9)				63 (43.8)			
Unknown*	97 (51.1)				85 (56.3)			
**Dental status**								
Good	66 (61.7)				63 (68.5)			
Needs treatment	138 (51.1)	1.218	(0.030–0.382)	0.025	124 (54.6)	1.472	(0.177–0.613)	<0.001
Edentulous	31 (28.4)				24 (28.6)			
Unknown*	14 (30.4)				12 (41.4)			

* Unknown data were not included in the calculations.

**Not included in calculations because of risk of sparse-data bias.

## Results

**Fig 1:**
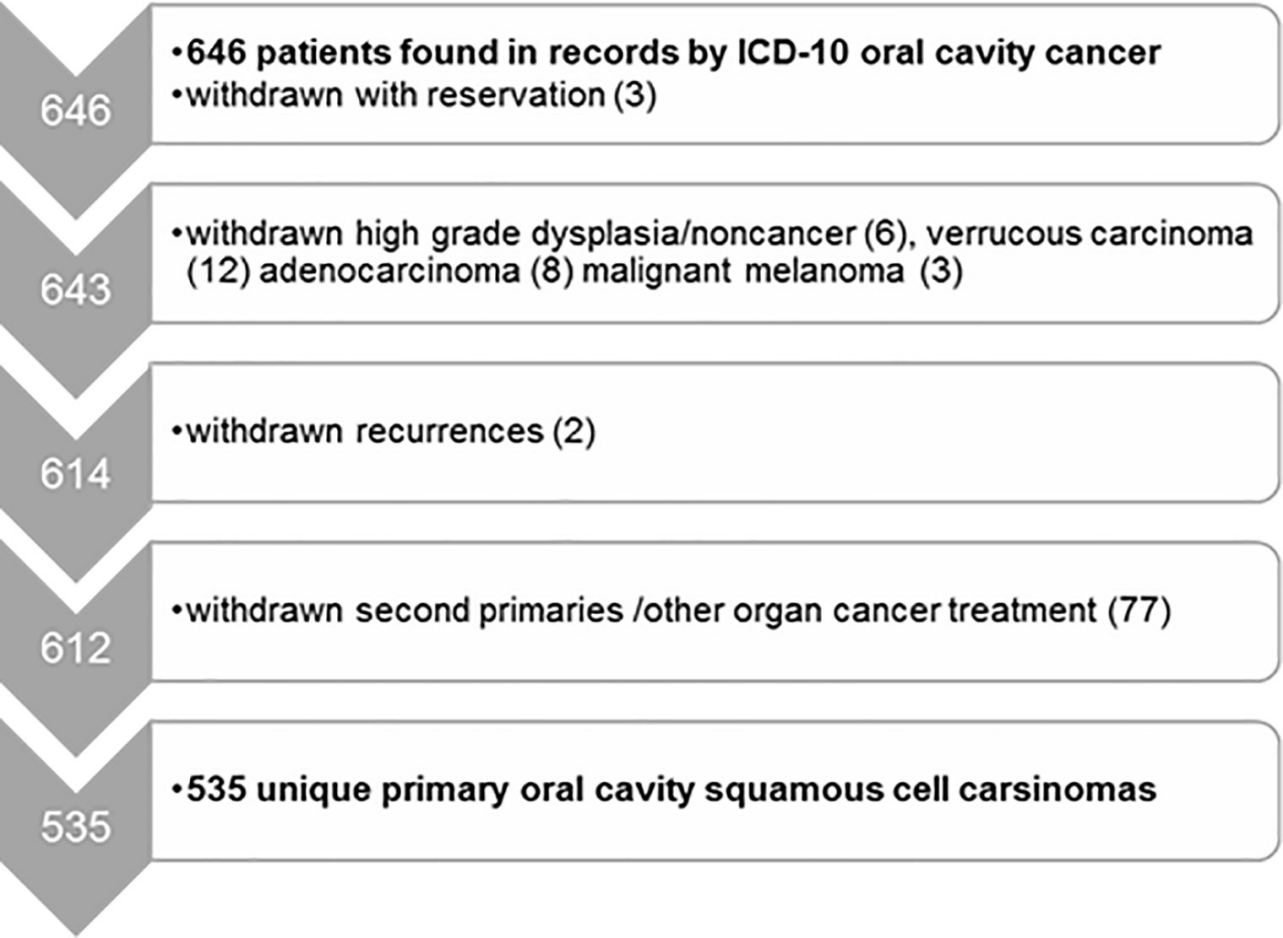
Flowchart outlining how we identified 535 unique primary treatment-naïve oral cavity squamous cell carsinomas in Norway in the time period 2005–2009.

Our study identified 646 patients with cancer in the oral cavity, of which 111 were excluded as specified in the flowchart in Fig 1, giving a final cohort of 535 patients diagnosed with primary treatment-naïve OCSCC.

The male: female ratio was 1.2, and the median survival follow-up time from end of primary treatment till death or last day of follow-up was 48 months (range 0–125 months).

### Clinicopathological characteristics

The clinicopathological characteristics are given in Table 1.

Median age at time of diagnosis for the whole cohort was 67 years (range 24–101 years) with few patients younger than 40 and older than 90 (13 and 11 cases, respectively). The median age at time of diagnosis for men was 64 years (range 25–101 years) compared to 72 years for women (range 24–96 years).

In 97% of the cases TNM staging was complete, and 93% of the cases were discussed in MDT meetings. There was no significant gender difference in either T or N status, and just slightly significant for stage.

T1 and T2 tumors constituted 53% of the cases, almost 11% were T3 tumors, and 33% were T4. According to the AJCC staging, 43% of the patients had stage I and II disease, 12% had stage III disease, and 42% had stage IV disease.

There was no significant gender difference in location of the primary tumor (Table 1). The mobile tongue was the most common tumor site, accounting for almost 50% of the cases for men and 40% for women. The second most common tumor location in men was floor of mouth while gingiva and alveolar mucosa were more frequent in women. Cancers in the mobile tongue were most often T1-T2 tumors, whereas gingiva and alveolar mucosa had more T4 tumors. Tumors of the floor of mouth were most often T2 and T4. There was no correlation between age group and location (p = 0.068, CI: 0.001–0.164). Only three patients had distant metastasis at time of diagnose, and no calculations were performed on this variable.

### Risk factors

Risk factors are listed in Table 2.

Smoking habits were recorded for 93% of the patients. There was a significantly lower proportion of never-smokers among male compared to female patients (14% vs. 34%), and 58% of the male patients were current smokers compared to 40% of female patients. Only two patients were recorded consuming Scandinavian snuff, but both were former smokers and recorded as such. Current smoking neither correlated with site of primary cancer (p = 0.175, CI: -0.025–0.141), nor with T status (p = 0.909, CI: -0.093–0.085) or with N status (p = 0.628, CI: -0.064–0.109).

Men consumed significantly more alcohol than women, with 11% of the men being heavy drinkers compared to less than three percent (2.5%) of the women (Heavy drinkers were included in the “drinker”-group). Alcohol consumption neither associated with site of primary cancer (p = 0.858, CI: -0.068–0.094), nor with T status (p = 0.522, CI: -0.111–0.054) or N status (p = 0.770, CI: -0.084–0.069). Of note, 35% of the EHR lacked information of alcohol consumption, and were excluded when analyzing for known risk factors.

More men than women were classified as smokers and drinkers according to the IRF classification, but the difference was not statistically significant. IRF correlated with T status (p = 0.001, CI: 0.067–0.237), but not with site of primary cancer (p = 0.265, CI: -0.035–0.125) or with N status (p = 0.856, CI: -0.060–0.081).

Half of the patients needed some form of dental therapy before treatment, whereas 40% had no need of dental treatment, of whom 20% were edentulous. The remaining 10% lacked information on dental status. There were more edentulous patients in the older than younger age groups (p<0.001, CI: 0.178–0.348). There was a significant correlation between dental status and gender, but when adjusting for age this difference was no longer present (p = 0.708, CI: -0.071–0.104). Patients with tongue cancer had significantly better dental status than patients with cancer in other oral sites (p = 0.002, CI: 0.039–0.213). In the five-year follow-up time recurrence was found in 95 (17.8%) of the patients within three years. Second primaries, defined as a new OCC more than three years after first presence of OCC, was found in 56 (10.5%) of the patients.

### Treatment

This study includes patients treated with both curative and palliative intention. Cancer treatment as described in the EHR are listed in Table 3. Palliative treatment was to some extent vaguely described and pooled.

For 69% (n = 386) of the patients the treatment was surgery, of whom 64% (n = 235) received postoperative RT as shown in Table 3. Very few of the patients had RT prior to surgery and there was no gender difference in this stratification of treatment (p = 0.215, CI: -0.171–0.026). Primary RT with or without chemotherapy was reported for 16%, and palliative treatment was effectuated for 11% (around six percent with RT, the rest with some debulking surgery or chemotherapy, vaguely described).

There was a significant difference in use of RT between age groups (p<0.001, CI: -0.334- -0.173). Women seemed to receive significantly less RT than men, but when adjusting for age there was no difference in use of RT between the genders (p = 0.381, CI: -0.049–0.124). Use of RT was significantly associated with higher T status (p = 0.008, CI: 0.027–0.207) and positive N status (p = 0.031, CI: 0.007–0.171) but not with site of tumor (p = 0.683, CI: -0.070–0.100).

### Survival

**Fig 2:**
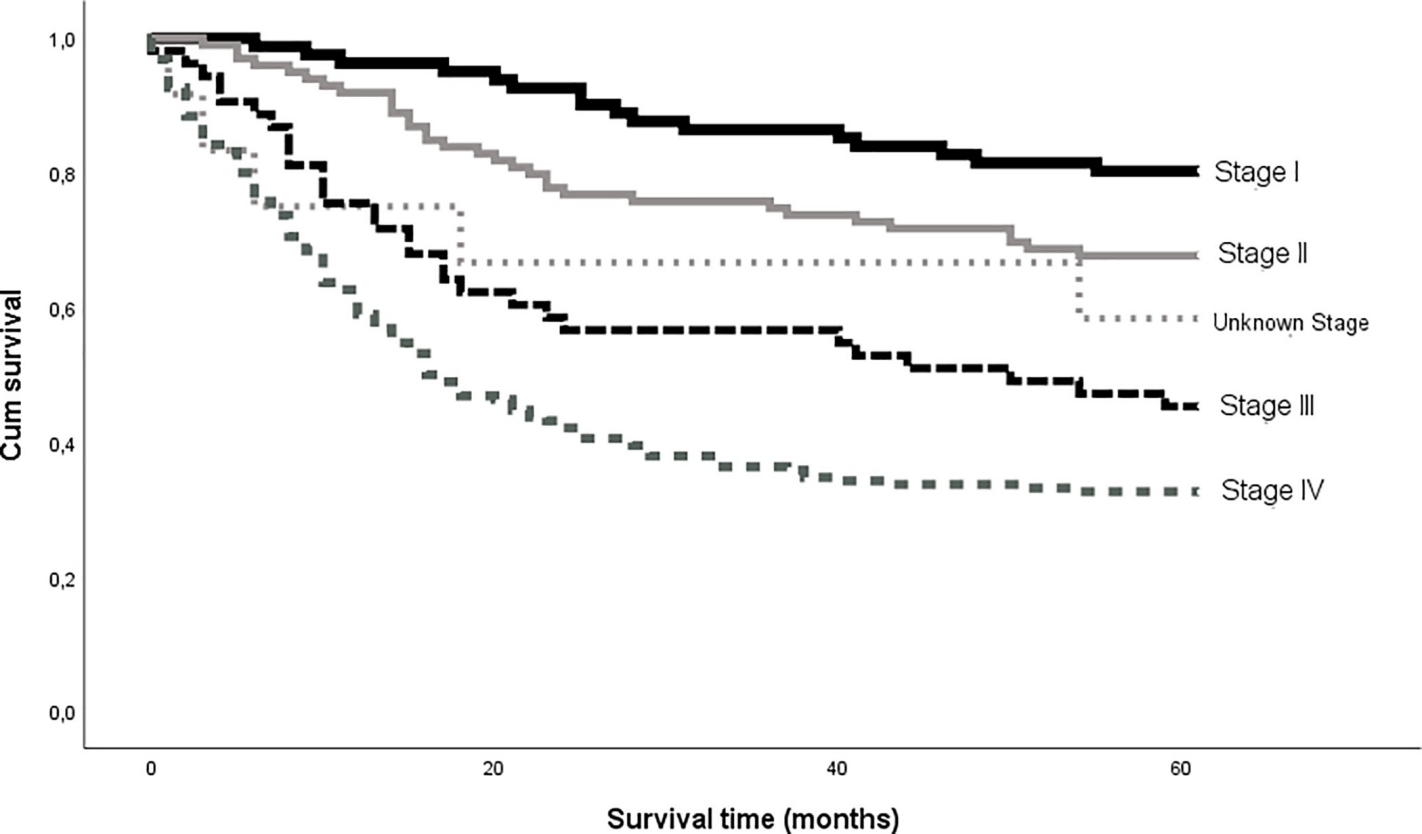
Kaplan-Meier curves show five-year disease-specific survival by stage in 435 patients diagnosed with primary treatment-naïve oral cavity squamous cell carcinoma in Norway in the years of 2005–2009.

Five-year overall survival (OS) was 47% for the cohort, and disease-specific survival (DSS) was 52% (225 of 435 patients). Five-year DSS was 80% for stage I, 68% for stage II, 45% for stage III, and 33% for stage IV (Fig 2).

When excluding patients given palliative treatment, the five-year OS and DSS for patients given treatment in curative intent, increased to 56% and 62%, respectively. The five-year DSS was then 80% for stage I, 68% for stage II, 51% for stage III, and 43% for stage IV (p<0.001, HR = 1.435, CI: 0.261–0.481). Tables 4 and 5 show calculations for five-year OS and DSS for the whole cohort compared to clinicopathologic characteristics and risk factors.

Age-groups, T status, N status, stage of disease, and dental status were all significantly associated with both OS and DSS, in univariate tests at p value <0.05 level (Tables 4 and 5). As stage is based on T status and N status, stage of disease was not included in multivariate analyses. Age-groups, T status, and N status were all independent predictors for OS in multivariate analyses (p = 0.001, HR; 1.487, CI: 0.288–0.517, p = 0.003, HR; 1.201, CI: 0.063–0.303 and p = 0.001, HR; 1.682, CI: 0.363–0.679). The same factors were independent predictors of DSS.

## Discussion

Our study is large, and includes a substantial number of well characterized patients with primary treatment-naïve OCSCC compared to other publications in this field. Many epidemiological studies present merged data for OCC and oropharyngeal cancer [1, 15, 18]. In our study around a quarter of the cases recorded as oral cavity cancers in the pathology archives and EHRs were oropharyngeal cancers and thus excluded from the study population. This suggests that there is a need to raise the awareness among both clinicians and pathologists of the importance of a correct anatomical description of the cancer site in patient medical records. Correct coding is crucial for proper cancer statistics and treatment. One should avoid using the ICD-10 diagnose C02.4 (tongue tonsils) as this can easily be misinterpreted as an oral location. Tumors arising in the root of the tongue is best coded as C01 (base of the tongue) recognized as an oropharyngeal location [38]. Separating oral cavity and oropharyngeal cancers is important as they are associated with distinct risk factors and also differ in primary treatment protocols, response to treatment, and survival rates.

The five-year DSS for our cohort was approximately 52%, which is in accordance with the global report from the review in 2009 of Warnakulasuriya et al. [9]. The five-year OS in our Norwegian cohort was 47%, which is somewhat higher than reported in a Danish cohort for the period 1980–2014 (44%)[13], but lower than in a Finnish study (61%). However, the Finnish study included only patients with OSCC of the tongue, treated with curative intent [5]. When we excluded patients given palliative treatment, the five-year OS and DSS increased to 56% and 62%, respectively. Although the OCSCC treatment in Norway is centralized to four university hospitals, some patients with small T1 tumors may have been treated at local hospitals without referral to the HN cancer centers, and would be missed from our cohort. Patients with T1 tumors have significantly better survival rates than patients with more advanced disease, and if some T1 tumors are missing in our cohort, this may have caused a negative shift in survival rate.

In the current study, 45% of the patients had cancer of the mobile tongue, which corresponds well to reports from previous studies [5–7]. The site and TNM classification are generally the most important prognostic factors for OCC [36]. In the present study, we found significantly higher survival for patients with low T status and stage, whereas the anatomical site of the tumor had no significant impact on survival. Number of recurrences or development of second primaries were in line with previous studies [5, 24].

There were more T1-T2 tumors in the tongue than in other locations, which may be caused by functions of the mobile tongue giving an earlier awareness of a tumor, along with the relative ease of self-inspection compared to other intraoral locations. The proportion of T4 tumors was higher in the gingiva and alveolar mucosa than in the other locations, which may reflect the short distance from the mucosa to the bone at these sites. Tumor involving the bone is classified as a T4 tumor irrespective of tumor size.

Stage I and II OCSCCs are often curable, thus early detection and treatment is of vital importance. In Norway, a large proportion of adults have regular dental examinations, and both dentists and dental hygienists are trained to examine the oral mucosa for malignant lesions. Still, we found 44% of the tumors to be size T3 and T4 at time of diagnosis, which could indicate a rapid growth of tumor. However, patients diagnosed with large tumors were more often edentulous or in need of dental treatment at time of diagnosis, suggesting that these patients did not seek dental care as frequently as those with smaller tumors. Older patients had larger T status, perhaps also reflecting later awareness of illness. It may also suggest that pain in the oral cavity, and symptoms such as changing diet and losing weight are regarded differently in elderly patients.

Globally, men have higher risk of OCC than women, and we found a male: female ratio of 1.2. This ratio is slightly lower than reported from a Danish and a US study (1.5 and 1.8 respectively) [13, 14], and slightly higher than reported in a Finnish study with 0.9 [5]. Tobacco and alcohol consumption have become more similar for men and women over the last three decades in Nordic countries compared to when the patients in our study were young [46]. The percentage of drinkers has also decreased both in Europe and in the US by approximately 10% since 2000 [47, 48].

Despite these changes in smoking and drinking habits, the incidence of OCC is rising. This suggests that other etiological factors are involved. For cancers arising in the oropharyngeal region, high-risk human papilloma virus (HPV) is considered to be an additional risk factor [49–51]. However, there is little scientific evidence to consider HPV as a risk factor for OCC, and the frequency of HPV positive SCC in the oral cavity is generally very low, with less than four percent in a Brazilian cohort [17] and less than 10% in a previous study from our group [16]. The use of Scandinavian snuff instead of cigarette smoking has increased tremendously over the last two decades in Norway, whereas cigarette smoking has decreased [52]. Future studies will reveal whether this influences the risk of OCC.

The choice of treatment was decided at MDT meetings for the vast majority of patients. This is according to current recommendations [35], and was a positive finding, as these patients were treated nine to 14 years ago when MDT meetings were less established than today. Cancer in the oral cavity is normally managed by surgical removal of the primary tumor, sometimes combined with neck dissection and/or RT, while chemotherapy is seldom used [28–34]. The same standard of treatment was also found in our cohort.

There are limitations to our study. It was not possible to specify the amount of tobacco use in pack-years or drinking units as this was a retrospective study. In as many as 35% of the EHR, the information of either smoking or drinking habits or both, were missing. Calculations must be evaluated with this perspective. The patient files stated present or past occupation, but not level of education. Level of education is interesting as a measure of cancer incidence in different socioeconomic classes. For a future prospective study one may recommend a systematic and accurate registration of socioeconomic status, smoking habits and alcohol consumption, as well as treatment modalities.

The Eighth Edition of the TNM classification has been introduced since this study was initiated, and in the new TNM classification tumor depth of invasion is included in the T classification of OCC, and this will influence determination of stage as well as prognosis [4].

## Conclusion

We present a study of a large cohort of 535 primary treatment-naïve OCSCC. Five-year DSS for the whole cohort was near 52%, and included patients receiving curative as well as palliative treatment. When extracting patients given treatment with curative intent, the five-year DSS increased to 62%. There was no gender difference in survival even though men on average were eight years younger than the women at the time of diagnosis. Patients with the smaller tumors have better prognosis, and this emphasizes the importance of early detection.

## Supporting information

S1 Dataset Supplementary information for 535 patients included in the Norwegian Oral Cancer (NOROC) study. (SAV)
